# Hierarchies of evidence applied to lifestyle Medicine (HEALM): introduction of a strength-of-evidence approach based on a methodological systematic review

**DOI:** 10.1186/s12874-019-0811-z

**Published:** 2019-08-20

**Authors:** D. L. Katz, M. C. Karlsen, M. Chung, M. M. Shams-White, L. W. Green, J. Fielding, A. Saito, W. Willett

**Affiliations:** 1 American College of Lifestyle Medicine, PO Box 6432, Chesterfield, MO 63006 USA; 2 The True Health Initiative, Derby, CT USA; 3 Yale Griffith Prevention Research Center, 130 Division St, Derby, CT 06418 USA; 4 Applied Clinical Nutrition and Global Public Health Programs, University of New England, 11 Hills Beach Rd, Biddeford, ME 04005 USA; 5 Department of Public Health and Community Medicine, Tufts University School of Medicine, 136 Harrison Avenue, Boston, MA 02111 USA; 6 Risk Factor Assessment Branch, Division of Cancer Control and Population Sciences, National Cancer Institute, 9609 Medical Center Drive, 4E204, Bethesda, MD 20850 USA; 7 Department of Epidemiology and Biostatistics, University of California at San Francisco School of Medicine, 550 16th Street, Second Floor, San Francisco, CA 94158 USA; 8 University of California Fielding School of Public Health, 650 Charles E Young Dr S, Los Angeles, CA 90095 USA; 9 Brown University School of Public Health, 121 S Main St, Providence, RI 02903 USA; 10 Harvard University T.H. Chan School of Public Health, 655 Huntington Avenue, Boston, MA 02115 USA; 11 Harvard Medical School, 25 Shattuck St, Boston, MA 02115 USA

**Keywords:** Strength of evidence, SOE, Systematic review, Lifestyle medicine, Lifetime effects, HEALM

## Abstract

**Background:**

Current methods for assessing strength of evidence prioritize the contributions of randomized controlled trials (RCTs). The objective of this study was to characterize strength of evidence (SOE) tools in recent use, identify their application to lifestyle interventions for improved longevity, vitality, or successful aging, and to assess implications of the findings.

**Methods:**

The search strategy was created in PubMed and modified as needed for four additional databases: Embase, AnthropologyPlus, PsycINFO, and Ageline, supplemented by manual searching. Systematic reviews and meta-analyses of intervention trials or observational studies relevant to lifestyle intervention were included if they used a specified SOE tool. Data was collected for each SOE tool. Conditions necessary for assigning the highest SOE grading and treatment of prospective cohort studies within each SOE rating framework were summarized. The expert panel convened to discuss the implications of findings for assessing evidence in the domain of lifestyle medicine.

**Results and conclusions:**

A total of 15 unique tools were identified. Ten were tools developed and used by governmental agencies or other equivalent professional bodies and were applicable in a variety of settings. Of these 10, four require consistent results from RCTs of high quality to award the highest rating of evidence. Most SOE tools include prospective cohort studies only to note their secondary contribution to overall SOE as compared to RCTs. We developed a new construct, *Hierarchies of Evidence Applied to Lifestyle Medicine* (HEALM), to illustrate the feasibility of a tool based on the specific contributions of diverse research methods to understanding lifetime effects of health behaviors. Assessment of evidence relevant to lifestyle medicine requires a potential adaptation of SOE approaches when outcomes and/or exposures obviate exclusive or preferential reliance on RCTs. This systematic review was registered with the International Prospective Register of Systematic Reviews, PROSPERO [CRD42018082148].

**Electronic supplementary material:**

The online version of this article (10.1186/s12874-019-0811-z) contains supplementary material, which is available to authorized users.

## Background

There is at present lively debate in the peer-reviewed literature regarding the nature of evidence supporting specific recommendations pertaining to nutrition [1, 2] and other components of lifestyle medicine [3]. Lifestyle medicine can be defined as the use of behavioral modifications in diet, exercise, sleep, stress, or substance use/exposure to prevent, treat, and potentially reverse lifestyle-related, chronic disease [4]. Such modifications may be implemented in clinical settings or more broadly as public health interventions, environmental changes to reinforce healthy default choices, or as online or distance-based interventions, but all with the intent to alter health behaviors among individuals.

Assessment of scientific evidence for a given question has evolved in academic publications from the presentation of an individual author’s conclusions into a formalized process [5–7] that involves conducting a systematic review of all available evidence within predetermined inclusion criteria. A common outcome of a systematic review is an assessment of “strength of evidence” (SOE) by the authors, starting with individual assessments of study quality followed by the use of a SOE grading tool to synthesize and summarize findings from all included studies. SOE is then often used to inform the next step in public health and clinical practice, writing practice recommendations, or assessing strength of recommendations [8, 9].

Evaluating SOE for research questions related to health behaviors of individuals is of high importance for public health professionals and clinicians focusing on behavioral modification as part of clinical practice. Interest in lifestyle medicine is rapidly expanding globally [10]. Lifestyle choices can have a major impact on burden of disease and premature death, even if the exact contributions of different components (exercise, diet, smoking, etc.) in the context of total lifestyle pattern are debated. Among the more frequent criticisms of lifestyle medicine is that conclusions and practice recommendations are not adequately informed by randomized controlled trials (RCTs) [11, 12]. Counter-arguments, noting the importance of other sources of evidence, have been published as well, at times in tandem [13, 14]. Thus, the importance of reliably interpreting relevant evidence about lifestyle choices has never been greater [15].

The majority of current systems for evaluating scientific evidence are well-suited to conventional medical treatment such as pharmacotherapy and discrete procedures. The movement towards evidence-based medicine (EBM) in recent years has emphasized the commonly accepted hierarchy of evidence and generally places results from RCTs above other study designs [16, 17]. While this is appropriate in many instances, RCTs are subject to specific biases and may not serve to address questions concerning the lifetime effects of health behaviors [18, 19].

Specifically, RCTs have methodological limitations that impede application to the investigation of longevity, overall vitality [20], compression of morbidity [21], and the lifetime [22–24] effects of diet, exercise, stress, sleep habits, and other lifestyle components, as well as ethical considerations depending on the research question. Such limitations have been examined in previous decades [18] and, more recently, in new publications highlighting the drawbacks of over-reliance on an RCT-centric model [19]. These limitations are particularly relevant in the context of developing healthcare practice guidelines for treatments that can withstand the challenges of real-world applications [16, 25]. Some such limitations of the RCT model include the following:

1. Cost constraints and challenges with adherence makes it difficult to randomize individuals to lifestyle interventions and maintain the prescribed behaviors for sufficient time periods (decades) to investigate the effects of such exposures on mortality or long-term morbidity [26, 27].
2. Blinding of the treatment group is only possible when the treatment is ostensibly similar to the placebo. While this is straightforward in drug trials, it is difficult at best, and often impossible when modifying health behaviors.
3. The generalizability of results in intervention trials to the broader population may be limited.

Some debate exists around differences in results seen between observational studies and RCTs. Depending on the research questions, evidence from observational cohort studies may be substantially more informative in drawing conclusions about overall SOE [28]. There may be a particular advantage in hybridizing evidence sources, recognizing that different evidence sources, from bench research, to intervention studies in humans, to observational epidemiology, make distinct contributions to understanding [17, 29, 30]. Therefore, it would be useful to have a method of evaluating SOE that is tailored to assessing lifestyle interventions and that can offer a more holistic assessment of evidence spanning diverse methods.

We conducted a methodologic systematic review of SOE tools to inform the answer to this question: When RCTs cannot, for whatever reason, serve as the primary evidence source, are there alternative assemblies of evidence that can be used to achieve comparable confidence in a given exposure-outcome relationship?

The research team was convened by the American College of Lifestyle Medicine (ACLM) in joint auspices with the True Health Initiative (THI) to (1) conduct a methodological systematic review of SOE grading tools in recent or current use to characterize which assemblies of evidence produce an evidence rating of highest strength, and (2) analyze the findings and their implications for potentially developing a new grading tool to evaluate SOE in the specific context of lifestyle medicine, where often good RCTs are not available or possible.

## Methods

The Preferred Reporting Items for Systematic Reviews and Meta-analyses (PRISMA) statement was followed in reporting this systematic review [31]. The protocol was prospectively developed and registered on the International Prospective Register of Systematic Reviews, PROSPERO, [CRD42018082148] [32, 33]. An expert panel (Additional file 1) in evidence-based medicine and its application to nutrition/lifestyle behaviors was convened to assess the findings and make recommendations.

### Search strategy

The search strategy was built in PubMed in consultation with a librarian and modified as needed for four additional databases: Embase, AnthropologyPlus, PsycINFO, and Ageline. The databases were searched for studies containing keywords related to either lifestyle or longevity. To identify only SOE tools in recent or current use, searches included studies published during the previous five years from the start of the project, from 01/01/2013–11/07/2017. There were seven exposures of interest related to lifestyle: diet, exercise, stress, social relationships/support, addiction(s), sleep, and genetic-based factors with potential for epigenetic modification. Additional search terms were included to restrict the scope of our literature search to papers related to avoidance of chronic disease: longevity, vitality, and healthy or successful aging. Keywords used in the search strategy are presented in Table 1. Search strategies were restricted to systematic reviews and meta-analyses conducted among humans and published in English, as the research team was not able to read or screen non-English papers. Umbrella reviews (systematic reviews of systematic reviews) were not included. To further focus on evaluation of evidence related to the lifetime effects of health behaviors and healthy aging, PubMed and PsycINFO searches were limited to studies in participants 65+ years of age. The complete search strategy for all five databases is presented in Additional file 2.

**Table 1:** Keywords used in the search strategy to identify systematic reviews and meta-analyses using relevant strength of evidence (SOE) tools

Keywords for exposures or interventions of interest (lifestyle)	Keywords for outcomes of interest (longevity)
• Diet	• Aging well
• Nutritional status	• Longevity
• Stress	• Successful aging
• Psychological	• Healthy aging
• Exercise	
• Social support	
• Family relations	
• Social isolation	
• Substance related disorders	
• Sleep	

### Inclusion/exclusion criteria

To identify relevant SOE tools in current or recent use, we included systematic reviews and meta-analyses of intervention trials or observational studies that both examined lifestyle medicine exposures and outcomes and evaluated SOE using a specified SOE tool. The inclusion and exclusion criteria applied in abstract and full-text screening are presented in Table 2. Included studies were required to contain only studies conducted in human adults and with at least one comparison group. Studies were excluded if they were conducted in children, healthcare workers, animals, or in vitro or if they only included single-arm trials (i.e., no comparison group). Studies were also excluded if they utilized any pharmaceutical- or supplement-based interventions, utilized genome-wide-association-studies (GWAS), or focused on research methods, validation of instruments or questionnaires, medical devices, or other assays. Additionally, given our focus on lifestyle medicine, studies were excluded if they examined research questions not relevant to lifestyle medicine (e.g., focused on the domains of injury severity, effectiveness of diagnostic tools or medical devices, or mechanistic questions that are tangential to lifestyle interventions or that were not clearly modifiable by lifestyle factors).

**Table 2:** Inclusion/exclusion criteria applied in abstract and full-text screening^1^

1) Criteria used in abstract screening	
Included exposures/interventions	
• Diet	
• Exercise	
• Stress or stress reduction	
• Social relationships	
• Addiction(s)	
• Sleep	
• Genetic-based factors	
Excluded exposures/interventions:	
• Pharmaceutical-based interventions	
• Studies on research methods (e.g., validation of a health questionnaire), medical devices, tests or other assays	
• Supplement interventions (e.g., micronutrient supplements, protein supplements) without accompanying lifestyle modifications	
• Genome-wide association studies (GWAS, i.e., an analysis comparing the allele frequencies of all available polymorphic markers in unrelated patients with a specific symptom or disease condition, and those of healthy controls to identify markers associated with a specific disease or condition)	
• Target population is children or healthcare workers	
2) Additional criteria used in full-text screening	
Included strength of evidence (SOE) tools that evaluated one of the following outcomes:	
• Longevity	
• Vitality and healthy or successful aging	
• Disease risk or disease incidence	
Excluded SOE tools addressed outcomes related to:	
• Disease prevalence	
• Injury severity	
• Efficacy or effectiveness of diagnostic tools, medical devices, or other assays	

^1^ The inclusion/exclusion criteria were used during abstract/full-text screening to identify potentially relevant articles that may include relevant SOE tools

### Study selection process

After merging results from all five databases and removing duplicates, all citations were title-screened by a single investigator [MK] to exclude in vitro, cell and stem cell studies, animal studies, and studies whose designs were clearly not a systematic review or meta-analysis, such as studies that used other study designs in the title (case report, randomized controlled trial, prospective cohort study, etc.) All studies with ambiguous titles were included at this stage of screening. All abstracts identified via the literature searches were then independently double-screened (independently screened by two different investigators) [MK, MSW, AS] using the inclusion and exclusion criteria (Table 2) via the open-source, online software Rayyan [34]. Full-text articles were retrieved for all abstracts deemed potentially relevant. Keyword text mining was performed to identify papers that mentioned text relevant to the use of a SOE tool [MK, MSW]. Full keyword search terms are presented in Additional file 3. Articles containing one or more of the keywords were then independently double-screened based on inclusion and exclusion criteria [MK, MSW]. All abstract and full-text screening conflicts were resolved through group discussion and final decisions reached by group consensus.

Additionally, the results from the systematic search process were complemented with manual searching on websites of major agencies recommended by the expert panel that conduct or commission systematic reviews. Agency websites were searched for officially adopted SOE tools [MK, MSW, AS]. A list of unique tools was compiled from the combination of the systematic and manual searches [MK].

### Data extraction

Data extraction forms were created and received approval from the entire research team prior to use. The information extracted included the following: date first published; purpose of the evaluation; intended audience; number of levels of SOE; the definition of the highest level of SOE; and the placement of cohort studies in the framework of SOE. All data extractors initially extracted 10% of the articles to pilot uniformity of extractions. For all remaining articles, each article was extracted by one investigator and reviewed and confirmed by a second [MK, MSW, AS]. Any disagreements were discussed among the research team and resolved via group consensus.

### Risk of bias (ROB) in individual studies

As this systematic review’s focus is on SOE grading systems related to lifestyle medicine outcomes and not studies’ specific lifestyle-related findings, ROB assessments were not conducted. However, if ROB assessments played a role in the included SOE grading systems, details were extracted.

### Data synthesis

Data were summarized in narrative form with regard to the conditions necessary for assigning the highest SOE grading (e.g., for assigning a grade “A” or level “1” rating). Next, the treatment of prospective cohort studies within each SOE rating framework was qualitatively summarized [MK, MSW, AS].

## Results

**Fig 1.**
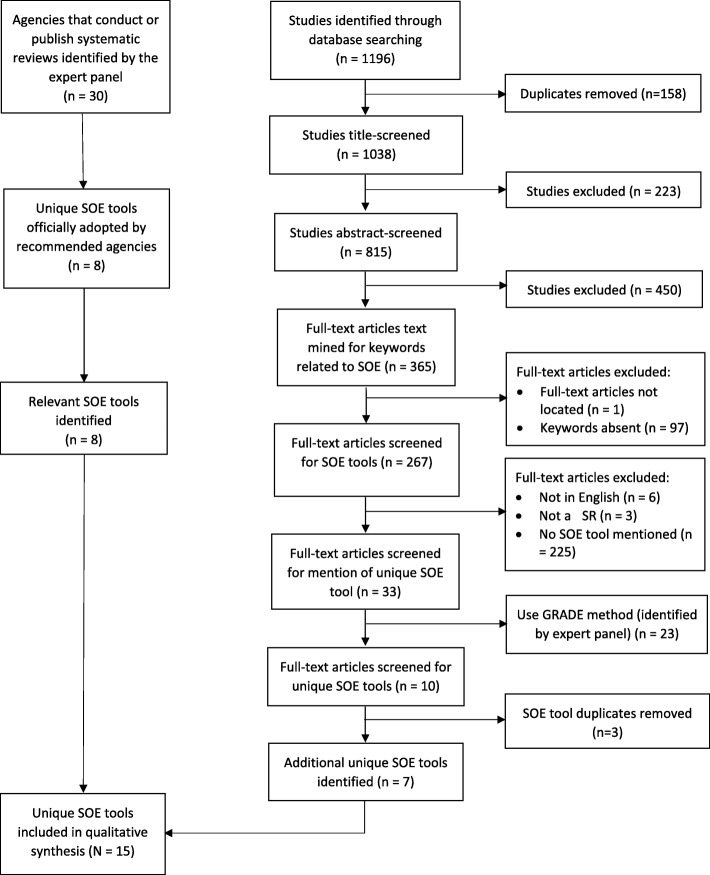
PRISMA flow diagram

The PRISMA flow diagram for study selection and exclusion is presented in Fig. 1. The manual search guided by the expert panel identified a total of eight unique SOE tools. The systematic search strategy identified a total of 1196 studies. Of these, 267 studies contained one or more relevant keywords. From these, a total of 33 studies mentioned using a specific SOE tool: 23 studies used Grading of Recommendations, Assessment, Development and Evaluation (GRADE) [35], which had previously been identified in the manual search, and 10 studies used a total of seven other unique SOE tools. Thus, a total of 15 unique tools are presented in Table 3.

All 15 tools rated SOE using three to five levels, with the exception of the US Food and Drug Administration (FDA) tool in reference to qualified health claims [36] (two levels). Of the 15 tools included, five were lesser-known methods defined by authors and primarily related to pain or physical rehabilitation and treatment [37–46]. The other 10 SOE tools were developed and used by well-known agencies and are applicable in a variety of settings [35, 36, 47–53]. Of these 10, four clearly require consistent results from RCTs of high quality to award the highest rating of evidence: GRADE [35], the FDA tool in reference to health claims for food products [36], the American College of Cardiology / American Heart Association Task Force on Practice Guidelines Levels of Evidence [54], and the Evidence-based Practice Center (EPC) method for grading SOE [51].

Four SOE tools describe more flexibility in the use of study design in determining ratings: the Community Preventive Services Task Force method [47] references study design and its “suitability for answering the research question;” the Grading System from the Academy of Nutrition and Dietetics [50] describes “studies of strong design for the question;”, the Johanna Briggs Levels of Evidence identifies different levels of evidence under the separate headings effectiveness, diagnosis, prognosis, economic evaluations, or meaningfulness [52], and the Oxford Centre for Evidence-Based Medicine (OCEBM) Levels of Evidence [53] uses a grid of five levels of evidence, where each level is specifically tailored to seven different kinds of research questions and supports a variety of combinations of quantity and quality of evidence depending on the specific research question.

With the exception of the OCEBM Levels of Evidence [53] specific mention of observational studies was made only in reference to their secondary contribution to overall SOE from RCTs, unless RCTs were methodologically flawed.

**Table 3:** Strength-of-evidence (SOE) rating tools

Name of SOE method, year	Audience and Purpose for Evaluation	Number of levels of SOE	Definition of the highest level of SOE	Placement of prospective cohort studies in the framework of SOE
Tools developed by major agencies, for application in a variety of domains				
Grading of Recommendations, Assessment, Development and Evaluation (GRADE), 2004 (35) from Cochrane Collaboration	Audience: Users of systematically developed clinical practice guidelines and recommendations (e.g., clinicians, patients, policymakers) Purpose: To provide a systematic and explicit approach to making judgments about the quality of evidence and the strength of recommendations	4 levels: - High - Moderate - Low - Very low	Randomized trials begin as high quality of evidence and observational studies as low quality of evidence. Randomized trials remain high if they provide: • Direct evidence without important study limitations • Low imprecision (i.e., large number participants and/or higher number of events with small confidence intervals), and • Low publication bias	Observational studies without special strengths constitute low quality evidence, though study characteristics can increase or decrease a study’s starting quality. The following strengths can increase the SOE rating from observational studies: • Strong evidence of association—significant relative risk (RR) > 2 (< or 0.5) based on consistent evidence from ≥2 observational studies, with no plausible confounders (+ 1), or • Very strong evidence of association—significant RR > 5 (< or 0.2) based on direct evidence with no major threats to validity (+ 2); • Evidence of a dose response gradient (+ 1); • Presence of all plausible residual confounding would have reduced the observed effect (+ 1) Note: Rigorous observational studies provide stronger evidence than uncontrolled case series.
Community Preventive Services Task Force (CPSTF), 2000 (47) No specific titled tool.	Audience: Community interventionists and clinical practitioners who need effectiveness recommendations for various treatments Purpose: To develop evidence-based, clinically effective recommendations for community-based interventions, various clinical treatments, and population-based interventions	3 levels: - Strong - Sufficient - Insufficient	3 possible paths to a “Strong” rating^a^: • ≥2 studies with “good” execution, “greatest” design suitability, and consistent effect sizes of “sufficient” size • ≥5 studies with “good” execution, “greatest or moderate” design suitability, and consistent effect sizes of “sufficient” size • ≥5 studies with “good or fair” execution, “greatest” design suitability, and consistent effect sizes of “sufficient” size	It is possible for a prospective cohort study to fulfill the requirements for the “Greatest” rating. Specific study designs are not rigidly placed within the framework; the suitability for answering the research question is assessed in reference to potential threats to validity.
US Preventive Services Task Force (USPST), 2012 (48) No specific titled tool.	Audience: Primary: primary care clinicians Secondary: consumer organizations, federal agencies, and other stakeholders involved in primary care delivery Purpose: To develop evidence-based recommendations about clinical preventive services and health promotion and evidence-based practice to improve the health of Americans	5 levels: - A: High certainty of substantial net benefit - B: High certainty of moderate net benefit or moderate certainty of moderate to substantial net benefit - C: Moderate certainty net benefit is small - D: Recommends against service, no net benefit or harm outweighs benefits - I: Insufficient evidence	• > 1 well-designed study • Consistent study results • Conducted in representative primary-care populations • Unlikely to be strongly affected by results of future studies	Prospective cohort studies and other specific study designs are not directly mentioned in this method. The highest level of evidence is described as coming from “... well-conducted studies in representative, primary care populations… [to]… assess the effects of preventive service on health outcomes...”
US Food and Drug Administration assessment of health claims for food products, 2003 (36) No specific titled tool.	Audience: Consumers of products with authorized or qualified health claims^b^ Purpose: To systematically evaluate the SOE for a proposed health claim,^b^ including both authorized and qualified health claims	2 levels: - (1): Authorized health claim (has significant scientific agreement among qualified experts) - (2): Qualified health claims- weaker scientific evidence must be accompanied by a disclaimer or be qualified in their wording (e.g., limited, very little, or highly uncertain scientific evidence)	• Studies with overall high methodologic quality rating • Results from intervention studies (as compared to observational studies) provide stronger evidence • Larger number of studies and sample sizes • Body of scientific evidence supports a health claim relationship for the US population or the target subgroup • Study results supporting the proposed claim have been replicated • Overall consistency in the total body of evidence showing a beneficial relationship	Observational studies: • Cannot be used to rule out the findings from well done intervention studies • Only included when findings are consistent with several RCTs • Any number of observational studies are trumped by several consistent RCTs • Hierarchy of evidence: Cohort design >nested case-control or case-cohort studies > case-control studies > cross-sectional studies > ecological studies and case reports
American College of Cardiology / American Heart Association Task Force on Practice Guidelines Levels of Evidence, 2005 (54)	Audience: Clinicians and researchers with an interest in cardiovascular health Purpose: To summarize SOE for the purpose of assigning classes of clinical practice recommendations	3 levels: - A: Data derived from multiple randomized clinical trials (RCTs) or meta-analyses. - B: Data derived from a single RCT or non-randomized studies. - C: Consensus opinion of experts, case studies, or standard of care	Multiple RCTs or meta-analyses of RCTs	Prospective cohort studies are not referenced in this method.
National Evidence Library Grading Rubric, 2015 (49)	Audience: Primary: US Dietary Guidelines Committee Secondary: Health professionals and the public who read the Dietary Guidelines for Advisory Committee Report Purpose: To summarize the SOE to make conclusion statements possible to inform policy (e.g., informing the Dietary Guidelines)	4 levels:* - Grade I: Strong - Grade II: Moderate - Grade III: Limited - Grade IV: Grade Not Assignable *Grading based on 5 elements: risk of bias; quantity of studies; consistency of findings; impact (directness of studied outcomes and magnitude of effect); generalizability to the US population of interest	• Bias - Studies of strong design free from design flaws, bias and execution problems • Quantity - Several good quality studies; large number of studies with sufficiently large sample size for adequate statistical power • Consistency - Findings generally consistent in direction, effect size or degree of association, and statistical significance with very minor exceptions • Impact - Studied outcome relates directly to the question and effect size is clinically meaningful • Generalizability - Studied populations, intervention and outcomes are free from serious doubts about generalizability	Prospective cohort studies are not directly mentioned in this method. The “risk of bias” component of the rubric mentions “studies of strong design” and “studies of weaker design for answering the question” but does not define them further.
Evidence Analysis Library® Methodology and Process Evidence Grading System from the Academy of Nutrition and Dietetics, 2016 (50)	Audience: Dietitians, clinicians, and researchers Purpose: To summarize the SOE for the purpose of making dietary recommendations	5 levels*: - I: Good/Strong - II: Fair - III: Limited/Weak - IV: Expert Opinion Only - V: Grade Not Assignable * Levels based on quality, consistency, quantity, clinical impact, and generalizability	• Quality: Strong study design for question; free from design flaws, bias and execution problems • Consistency: Findings generally consistent in direction and size of effect or degree of association, and statistical significance with minor exceptions • Quantity: ≥1 good quality studies with large sample sizes; studies with negative results have sufficiently large sample size for adequate statistical power Clinical impact: Studied outcome relates directly to the question; size of effect is clinically meaningful; large, statistically significant difference • Generalizability: Studied populations, interventions and outcomes are free from serious doubts about generalizability	Specific study designs are not mentioned or explicitly tied to a specific level of evidence. The quality rating for the highest level of evidence specifies “studies of strong design for the question.”
Evidence-based Practice Center (EPC) method for grading SOE, 2009 (51)	Audience: Clinicians, researchers, and other health professionals Purpose: Summarize SOE for the purpose of guiding clinical practice recommendations and to improve the quality of healthcare	4 levels: - High - Moderate - Low - Insufficient	Evaluation is based on 5 required domains and, where appropriate, 3 more optional domains: 5 required domains: • Study limitations/risk of bias: Low • Directness: High • Consistency: High • Precision: High • Reporting bias: Low 3 optional domains: • Dose-response association: Present • Uncontrolled confounding that can diminish an observed effect: Low • Strength of association (i.e., large magnitude of effect): High	• Domain and total SOE grading should be done separately for RCT evidence and observational study evidence. • Initially, RCTs start with a provisional high SOE grade and observational studies with a provisional low SOE grade. • These grades are adjusted as stronger or weaker based on study limitations or other factors.
Joanna Briggs Institute Levels of Evidence*, 2013 (52) *No longer in current use, organization recently switched to using GRADE; grading for research questions of *effectiveness* is presented here as the most relevant domain for lifestyle medicine-type interventions	Audience: Researchers Purpose: Summarize the SOE	4 levels under *effectiveness* heading:* - Level 1: Experimental Designs - Level 2: Quasi-Experimental DesignsLevel 3: Observational-Analytic Designs - Level 4: Observational-Descriptive Studies - Level 5: Expert Opinion and Bench Research Each level contains sub-levels	Effectiveness Level 1 categories are defined as follows: • Level 1.a – Systematic review of RCTs • Level 1.b – Systematic review of RCTs and other study designs • Level 1.c – RCTs • Level 1.d – Pseudo-RCTs	Prospective cohort studies* appear only in Level 3 categories (not Levels 1 or 2) • Level 3.a – Systematic review of comparable cohort studies • Level 3.b – Systematic review of comparable cohort and other lower study designs • Level 3.c – Cohort study with control group • Level 3.e – Observational study without a control group) “Inception cohort studies” do appear in Level 1 under *prognosis* heading
Oxford Centre for Evidence-Based Medicine (OCEBM) Levels of Evidence, 2011 (53)	Audience: Physicians Purpose: To provide traditional critical appraisal and summarize SOE for clinicians and patients to quickly guide decisions to clinical questions	5 levels: - Level 1 - Level 2 - Level 3 - Level 4 - Level 5 Each of the 5 levels are defined separately for each of the 7 clinical questions.	Level 1 evidence definitions for each of seven clinical questions: • 1. How common is the problem? Local and current random sample surveys (or censuses) • 2. Is this diagnostic or monitoring test accurate? (Diagnosis) Systematic review of cross-sectional studies with consistently applied reference standard and blinding • 3. What will happen if we do not add a therapy? (Prognosis) Systematic review of inception cohort studies • 4. Does this intervention help? (Treatment Benefits) Systematic review of randomized trials or n-of-1 trials • 5. What are the COMMON harms? (Treatment Harms) Systematic review of randomized trials, systematic review of nested case-control studies, n-of-1 trial with the patient you are raising the question about, or observational study with dramatic effect • 6. What are the RARE harms? (Treatment Harms) Systematic review of randomized trials or n-of-1 trial • 7. Is this (early detection) test worthwhile? (Screening) Systematic review of randomized trials	Prospective cohort studies ^c^ appear in the following clinical questions: • 3. What will happen if we do not add a therapy? (Prognosis) o Level 1: Systematic review of inception cohort studies o Level 2: Inception cohort studies o Level 3: Cohort study or control arm of randomized trial. Level may be graded down on the basis of study quality, imprecision, indirectness (study PICO does not match questions PICO), because of inconsistency between studies, or because the absolute effect size is very small; Level may be graded up if there is a large or very large effect size.) • Does this intervention help? (Treatment Benefits) o Level 2: includes observational study with dramatic effect o Level 3: Non-randomized controlled cohort/follow-up study • 7. Is this (early detection) test worthwhile? (Screening) o Level 3: Non-randomized controlled cohort/follow-up study
Author-defined / lesser-known methods				
Modified form of coding system, 2000 (37)	Audience: Researchers Purpose: To evaluate SOE related to correlates of physical activity in children and adolescents	3 levels: - Association (either positive or negative): 60–100% of studies reviewed support association - Indeterminate: 34–59% of studies reviewed support association - No association: 0–33% of studies reviewed support association	Highest level is achieved when 60% or more of studies (regardless of design or total N) reviewed have a consistent positive or negative association.	Study design is not referenced in this method. All studies’ results would count equally towards SOE score; no instructions are given with respect to weighting of different study designs.
Topic-specific SOE rating system for evaluating research on back pain, 1996 (38, 39)	Audience: Researchers and clinicians with an interest in back pain Purpose: To guide clinical practice guidelines for back pain	4 levels: - Strong - Moderate - Limited - No evidence	Multiple high-quality RCTs with consistent positive outcomes	Prospective cohort studies are not referenced (i.e., they are not relevant to this kind of evaluation).
Best evidence synthesis: a rating system based on a best-evidence synthesis used previously for PA interventions, 1995 (40–43)	Audience: Researchers Purpose: To summarize the SOE	4 levels: - Level 1: Strong - Level 2: Moderate - Level 3: Limited - Level 4: No evidence	Multiple RCTs of high quality with consistent positive results.	Prospective cohort studies are not referenced (i.e., they are not relevant to this kind of evaluation).
Criteria for determining level of evidence in meta-analyses of RCTs for walking training in stroke, 2008 (44)	Audience: Researchers and clinicians Purpose: To determine SOE in relation to rehabilitation after stroke	4 levels - High - Moderate - Low - No evidence	At least 2 high-quality RCTs with similar results	Prospective cohort studies are not referenced (i.e., they are not relevant to this kind of evaluation)
Overall SOE, 1999 (45, 46)	Audience: Researchers and clinicians Purpose: To predict the onset of functional status decline in people without initial functional status impairment	4 levels: +++ [Strong] ++ [Moderate] + [Limited] (+) [Weak]^d^	• Evidence in > 3 “high quality studies” with a consistent positive or negative association • Analyses have no identified methodological limitations • Studies exclude individuals with functional status impairment at baseline • Studies report a significant positive association between risk factor and functional status decline in people	Study design is not referenced in this method. All study designs can count equally in the SOE score, provided they were not identified as having methodological limitations (so were therefore classified as “appropriate”); no instructions are given with respect to weighting of different study designs.

^a^Sufficient effect sizes are defined on a case-by-case basis and are based on Task Force opinion. Each study is categorized as having good, fair, or limited quality of execution based on the number of limitations noted, studies with 0–1, 2–4, and 5 or more limitations are categorized as having good, fair, and limited execution respectively. The suitability of study design has 3 levels: Greatest, Moderate, and Least. Greatest: Concurrent comparison groups and prospective measurement of exposure and outcome; Moderate: All retrospective designs or multiple pre or post-measurements but no concurrent comparison group; Least: Single pre and post-measurements and no concurrent comparison group or exposure and outcome measured in a single group at the same point in time.

^b^Health claims characterize the relationship between a substance (such as a food or food component) and a disease or health-related condition.

^c^Prospective cohort studies are mentioned in question 5 (What are the COMMON harms?) and question 6 (What are the RARE harms?). However, they are not described in this table because of their limited relevance to lifestyle medicine interventions, which typically do not cause the harmful side effects seen in pharmaceutical treatment trials.^d^[descriptors] added for this table

### Conceptualization of SOE approach specific to lifestyle Medicine

Upon completion of the systematic review, the expert panel convened to discuss the findings. The results confirmed that the following methodological elements within existing SOE tools in recent use are lacking:

1. Criteria to evaluate exposure-outcome relationships examined over years/decades/lifetimes
2. Criteria to evaluate behaviors/exposures used in lifestyle medicine that may not allow for randomization or blinding (e.g., smoking, long-term dietary patterns, etc.)
3. Guidance to synthesize findings from diverse study designs, except to prioritize RCTs over observational studies.

To address these issues, the Hierarchies of Evidence Applied to Lifestyle Medicine (HEALM) investigators enumerated the particular contributions of diverse research methods into a complete understanding of exposure/treatment effects, as shown in Table 4.

**Table 4:** Contributions of evidence from the major categories of research approaches

Research Method	Unique contribution to understanding
Basic science	Mechanisms of action
Intervention studies in humans / RCTs	Reliable attribution; control of bias, confounding
Observational epidemiology; large and diverse population-based samples	Effects at scale
Observational epidemiology; long time periods	Duration of effects

Based on this simple framework, a new method for selecting the criteria by which SOE can be assessed was developed, titled *Evidence Threshold Pathway Mapping* (Table 5). It is intended to formalize and make explicit the decision process of which method or tool to use to evaluate SOE. With strength defined operationally as the relevant “threshold” value for some level of confidence, this potential methodologic innovation offers an opportunity to identify the assemblies of evidence that are most appropriate for a given research question, such as change in intermediate risk factors, short-term alleviation of disease symptoms, long-term improvement in diagnosed disease, or long-term prevention. The basic propositions underlying Evidence Threshold Pathway Mapping are that (a) different methods of research are best suited for making different yet complementary contributions to the overall weight of relevant evidence, and (b) different assemblies of evidence can produce the same aggregate strength or confidence. We recognize that in the absence of RCT data for treatment effects, certainty about treatment effects from other types of evidence may be more limited; thus, there is a basis to weight the contributions of RCTs preferentially. However, other types of evidence may still offer a spectrum of certainty or additional context for understanding.

**Table 5:** Evidence Threshold Pathway Mapping

Is the question definitively addressable with RCTs?^1^ YES if: The outcome of interest would be measurable in < 5 years, subjects can ethically be randomized, a control group is plausible and ethical, blinding is potentially possible, a sample size of < 10,000 would provide adequate statistical power If YES, have RCTs been conducted? ➔(1) If YES, then use GRADE^2^ ➔(2) If NO, then use an alternative tool, consider OCEBM^3^ NO if: A duration > 5 years adherence to the intervention is required, randomization is not plausible or ethical, exposure of interest is the cumulative, lifetime effect of health behaviors. ➔ (3) Consider HEALM^4^	

^1^ RCTs: randomized controlled trials

^2^ GRADE: Grading of Recommendations, Assessment, Development and Evaluation;

^3^ HEALM: Hierarchies of Evidence Applied to Lifestyle Medicine

^4^ OCEBM: Oxford Center for Evidence Based Medicine

Also implicit in this approach is the contention that various research methods serve different objectives related to evidence about a causal pathway. Bench science and animal model studies are most often used to establish clear and decisive evidence of mechanisms but cannot establish in vivo effects in humans [29]. Controlled intervention studies, and most notably RCTs, are used to establish attribution with confidence, while minimizing bias and controlling for both known and unknown confounders [17]. However, RCTs are not always ethically or practically feasible and they are demanding to implement at the population level, or over time periods relevant to lifetime vitality [30]. They also can introduce sampling bias that may greatly limit generalizability or external validity. Observational epidemiology, notably prospective cohort studies and even ethnographic studies, can readily assess associations at scale and over extended time periods (decades), but these are subject to bias including sampling bias, residual confounding, and they lack the capacity of RCTs to assign attribution with clarity [30].

Accordingly, evidence is strongest when the unique contributions of these diverse methods are synthesized. Making conclusions by drawing from a diversity of evidence sources can potentially allow for confidence in study design methods from one type of research, confidence in attribution from another type, confidence in effects at scale from yet another, and confidence in effects over extended timelines from another still. This amalgamation of complementary evidence is especially important when research questions cannot be readily answered by one study design alone (e.g., What dietary pattern produces the best health outcomes over a lifetime?) [55]. Such considerations are a subject of active discussion in nutrition research [56, 57].

Thus, we introduce a new construct- *Hierarchies of Evidence Applied to Lifestyle Medicine* (HEALM) shown in Table 6, to illustrate means of assessing SOE in future systematic reviews within the domain of lifestyle medicine when the use of GRADE or another SOE tool is not appropriate.

**Table 6:** Hierarchies of Evidence Applied to Lifestyle Medicine (HEALM) Strength of Evidence (SOE) Approach*

HEALM contains three scoring** levels of SOE: Grade A (Strong/decisive); Grade B (Moderate/suggestive); Grade C (Insufficient/inconclusive)	
As in other SOE evaluation methods, included studies’ methodological quality and risk of bias should be graded prior to assessment with HEALM established tools for rating individual study quality. Two examples are Cochrane’s Risk of Bias Tool^54^ for randomized controlled trials (RCTs) and the Newcastle-Ottawa Tool^55^ for cohort and case-control studies. Q1: Are there established mechanisms of action? (a plurality*** of evidence from bench science and animal models) Yes = 2 Uncertain*** = 1 No = 0 Q2: Are there intervention studies in people that provide evidence of causality/attribution? (a plurality*** of high-quality intervention trials, randomized controlled trials, interim measures, and surrogate markers as outcomes) Yes = 3 Uncertain = 1 No = 0 Q3: Are there observational studies to establish generalizability to large, populations? (a plurality*** of high-quality evidence from large prospective, cohort studies) Yes = 2 Uncertain = 1 No = 0 Q4: Are there observational studies to support effects over time periods measured in decades, lifetimes, or generations? (a plurality*** of evidence from high quality, long-term observational studies; retrospective cohort studies; ethnography; transcultural studies) Yes = 2 Uncertain = 1 No = 0	
*The HEALM tool is presented here to illustrate potential approaches to scoring evidence across research categories; it does not represent the single, specific approach recommended by the project expert panel on the basis of a formal process consensus process. **Scoring Answers to scoring questions should be based on expert consensus in evaluating available evidence. Evidence is conclusive when it can be identified as sufficient in quantity and quality, and consistent in findings, fostering clear consensus among experts. This would generally mean a replicated finding, and consistent effects among a clear plurality** of high quality, related publications.Evidence is uncertain when studies are few, small, poor quality, or conflicting- but generally suggestive of a particular finding. While expert consensus is critical in evaluation, a framework to inform discussion based on quantitative criteria used in previous umbrella reviews^56^ is suggested: 1. Total sample and number of cases of included studies 2. Significance of association based on *p*-values (highly significant defined as *p* < 0.0001 vs. nominally significant defined as *p* < 0.05) and confidence intervals that exclude vs. include the null value 3. When considering studies that include meta-analyses, a target threshold of 1000 cases, no evidence of small-study effects or excess significance bias, a 95% prediction interval excluding the null value and no large, unexplained, between-study heterogeneity (I^2^ < 50%) Grade A: Strong evidence = ≥7 (this would require decisive evidence in all other categories, AND at least suggestive evidence from intervention trials in people; OR- strong evidence from intervention trials in people, and decisive evidence in other two categories; OR strong evidence from intervention trials, decisive evidence in any other category, and suggestive evidence in the remaining two. Lends a primacy to RCT evidence but allows for strong evidence even with nothing more than suggestive evidence in intervention trial category. Grade B: Moderate/suggestive = 5 or 6. Achievable with decisive intervention trial evidence, and strong evidence in ANY other category. OR, strong evidence in all categories other than intervention trials. Grade C: Insufficient/weak/C = < 5 **Plurality may vary depending on the total number of existing studies conducted on a particular research question and must be determined on a case-by-case basis. For example, three consistent studies from a variety of study design with no opposing studies may constitute a plurality. Were there to be opposing studies the target number would be more than three. A clear numerical plurality of studies but with overall poor quality may constitute a rating of “Uncertain”.	

HEALM incorporates the variety of sources of evidence available and synthesizes their contributions into one rating. It is important to note that the method described in Tables 5-6 suggests one specific framework for handling a set of considerations around SOE. Alternative ways of handling such considerations including using a conventionally defined tool such as GRADE, not utilizing a predetermined scoring system, or uniquely adapting an existing tool to the research question being asked. We introduce Evidence Threshold Pathway Mapping and HEALM to illustrate one example of a suitable, customized approach for researchers in lifestyle medicine that can be applied, tested, and validated in practice. The proposed approach for evaluating SOE is informed by the flexibility and specificity presented in OCEBM [53]. HEALM adapts this approach to the specific exigencies of lifestyle medicine, while placing an emphasis on the alignment of research methods with specific questions related to causal pathways. To identify when use of such a tool might be appropriate, we suggest employing Evidence Threshold Pathway Mapping (Table 5) to map the pathway for evidence evaluation along the branches of a simple decision tree. For example, this process produces a suggestion to use the HEALM tool for all research questions concerning lifetime cumulative effects of specific health behaviors, as lifetime effects cannot be assessed in < 5 years. However, it suggests using GRADE [35] for other questions that are feasibly answered with RCTs.

## Discussion

Lifestyle behaviors are among the leading determinants of health outcomes, with non-communicable disease causing nearly three-quarters of death globally [58]. Dietary patterns have recently risen to the very top of this list [15], and there is intense debate about the strength and reliability of pertinent evidence [1–3]. The majority of current systems for evaluating scientific evidence are well-suited to evaluating pharmaceutical approaches to managing disease, but currently a system for evaluating SOE particular to lifestyle medicine does not exist.

Assessment of SOE requires grading the methodological quality and ROB of individual included studies, assessing the consistency and internal validity of studies addressing a specific research question, and forming conclusion statements. Such SOE conclusions can thus inform the discussion on the weight of evidence, informed by multiple studies providing for external validity or generalizability to various populations, settings, and circumstances.

Evidence Threshold Pathway Mapping contends that the same level of confidence, and the same strength of evidence, can be achieved by a variety of assemblies of evidence. The approach respects the unique value of RCTs in establishing attribution and does not assume RCTs are interchangeable with other study designs. Rather, Evidence Threshold Pathway Mapping acknowledges that RCTs may be precluded for various reasons with regard to a given outcome and that other complementary evidence should be considered. Even then, such trials may contribute to understanding by assessing attribution with use of interim measures, and/or surrogate markers. This method of identifying the SOE approach used for evaluation based on the nature of the question being asked is informed by the approach taken in the OCEBM tool [53], which tailors SOE evaluation for different types of research questions.

HEALM, derived from application of the Evidence Threshold Pathway Mapping approach, is one unique, potential approach organized to frame discussion of existing evidence available to answer specific research questions relevant to lifestyle medicine when existing tools such as GRADE are not viable options (i.e., the question is not fully addressable through RCTs). The scoring, similar to other SOE tools, relies on expert consensus, but is also informed by quantitative scoring considerations used in umbrella reviews [59] to evaluate results from multiple meta-analyses. While grading SOE does not necessarily mean meta-analyses will always be conducted, a quantitative framework to guide discussion will lead to greater consistency of results. HEALM defines categorical levels of SOE, as is conventionally done when evaluating evidence. However, it should be noted that such categories are derived from a continuum of SOE and that the value of the categories is to increase the utility of the tool for communicating findings. The intended purpose of HEALM is to evaluate SOE, which can then be used to develop strength of recommendation-based practice statements. The construct first introduced here may gain traction as is; it may be revised and refined by others; or it may be replaced outright if an alternative metric serving the same goals performs better.

The need for innovation in SOE assessment is in part because the RCT holds a position of relative primacy in the adjudication of medical evidence. Arguments favoring reliance on RCTs rightly invoke the merits in this methodology, namely defense against diverse kinds of bias, and protection against confounders both known and unknown [17] thus prioritizing internal validity. There are, however, diverse and valid concerns with the limitations of RCTs [30] in achieving external validity.

Also of concern are the cases in which observational and intervention trial results appear to be in conflict with one another. In some cases, RCTs may be testing different hypotheses than observational studies, and conclusions from one investigation may not be generalizable to all populations. For example, a review analysis on the use of hormone replacement therapy (HRT) among women in the Women’s Health Initiative affirms the consistency of findings across observational and intervention data if the age at time of starting HRT is considered [60].

A recent Cochrane systematic review concluded such differences are likely not due to differences in study design alone; rather, RCTs and observational studies tend to produce similar effect sizes for a range of health outcomes and disagreements are likely due to other study characteristics [61] such as testing different hypotheses [60] or duration of follow-up. While there are examples of RCTs that document outcomes after several years of follow-up post- intervention [62, 63], the challenges of adherence [27] severely limit feasibility of continuous interventions over decades. To the authors’ knowledge, there are no RCTs that have successfully and continuously implemented an intervention, especially one with a potentially small effect size, for the decades necessary to test “lifetime” effects. Thus, the prevailing impression that results from RCTs are *consistently* superior may be exaggerated, with the benefits and risks of hormone replacement therapy providing an example of the partial contributions to understanding made possible by both RCTs and observational cohort studies [64–67].

In contrast, there are clear cases in which observational studies offer a superior method of evaluating questions concerning the cumulative, lifetime effects of lifestyle practices. A key example of such trials whose recruitment is designed to maximize the number of endpoints is the Alpha-Tocopherol, Beta-carotene Lung Cancer Prevention (ATBC) Study which targeted male smokers [28]. In capturing hard endpoints such as cancer and cancer-related mortality, short-term RCTs would be of insufficient duration to see the outcome of interest, as well as being impossible to implement with exposures like smoking for ethical reasons.

The HEALM tool scores evidence, lending particular weight to RCTs for the clarification of causal effects and attribution. The tool, however, allows for rating evidence as strong even if RCT data are not more than suggestive, provided evidence from all other complementary research approaches are decisive and aligned. More importantly, short-term evidence from RCTs, or focus on isolated biomarkers, absent any suitably long-term data addressing hard outcomes would not score as “strong” in the realm of lifestyle medicine because of the great potential divide between short and long-term effects. As an example, many serious infectious diseases lower weight and blood lipids; such “favorable” trends in biomarkers are obviously not indicative of beneficial health effects in the long term. This adaptation of established approaches readily accommodates the imperative of judging the impact of lifestyle practices on health outcomes over the full human life span.

The strength of this study was to take an approach of a methodological systematic review to capture existing and recently used SOE tools, thus ensuring that a new method proposed would offer a novel contribution to address current methodological gaps. Limitations of this study included the focus in the search strategy on healthy aging as an outcome, rather than risk for specific chronic diseases. The search strategy was constructed in alignment with the target outcomes of lifestyle medicine practice (healthy aging, as opposed to chronic disease), and inclusion of all major chronic disease outcomes would not have been practical due to the large number of search results. Additionally, the search strategy was limited to systematic reviews of studies conducted among those ≥65 years, not because lifestyle medicine is only relevant for older populations, but because this focused the search strategy to identify studies in the domain of longevity. SOE tools used in these contexts would be potential best matches for evaluating evidence concerning other lifestyle medicine-type questions. However, manual searching for SOE tools based on expert panel recommendations augmented the systematic review results to the degree that all major tools known by the expert panel are included in our results.

Finally, the HEALM construct is dependent on conclusions about the “plurality” of evidence from distinct research methods. Other than results produced from systematic review of meta-analysis, there is no universal standard for sufficient or sufficiently consistent evidence to establish the veracity of a given causal pathway or weight of the evidence for a given research topic. Even meta-analyses and systematic reviews fail to reach this standard, because in “crowded” research domains more than one such study is common and they may conflict with one another. The Community Preventive Services Task Force (CPSTF) [47] provides some guidance on assigning strength of recommendations based on SOE conclusions by suggesting that inconsistent evidence should lead to separate recommendations for specific populations, and that no conclusions should be reached in the case of conflicting evidence. However, this guidance does not provide a framework for synthesizing strength and weight of evidence more broadly. Further, a limitation of HEALM is that it utilizes categories to assign relative levels of confidence, though this limitation is common to existing SOE tools.

The problem of establishing an operational definition for the “weight of evidence,” or a decisive plurality of studies, is in no way specific to lifestyle medicine. This is a generic challenge pertaining to all assessments of overall evidence, and thus deemed beyond the scope of this particular effort. This group simply notes the importance of this issue, and its pertinence to both Evidence Threshold Pathway Mapping and HEALM. This paper invites attention to the matter and highlights the opportunity to fortify operational definitions in this area.

This project was commissioned with a preferential focus on lifestyle medicine, but the implications apply broadly to public health. Lifestyle practices and exposures- dietary patterns, physical activity patterns, sleep patterns, tobacco and alcohol exposures, psychological stressors, social connections- while uniquely emphasized in lifestyle medicine (4), pertain to all fields of medicine and public health and to all health professionals.

Future research should test application of Evidence Threshold Pathway Mapping and HEALM by conducting systematic reviews on specific research questions in the domain of lifestyle medicine. The HEALM construct should evolve, informed by research in which it is applied.

## Conclusion

SOE tools in current use are generally poorly suited to long-term effects of lifestyle choices such as diet, exercise, sleep, and stress. Evidence Threshold Pathway Mapping, a method for identifying multiple assemblies of evidence to achieve a given grade, extends the robust assessment of evidence to a wider array of questions important to medicine and public health. HEALM is proposed as one example of a tool specifically adapted to questions in lifestyle medicine and nutrition. Application, testing, and validation of the performance of HEALM and consideration of its relevance to this domain of medicine are encouraged.

## Additional files

Additional file 1: Expert panel (DOCX 13 kb)

Additional file 2: Search strategy (DOCX 17 kb)

Additional file 3: Keywords used in text mining (DOCX 13 kb)

## Data Availability

Data sharing is not applicable to this article as no datasets were generated or analyzed during the current study. All data used in this systematic review are accessible via the papers referenced in the manuscript.

## Abbreviations

ACLM: American College of Lifestyle Medicine
ATBC: Alpha-Tocopherol, Beta-carotene Lung Cancer Prevention
CPSTF: Community Preventive Services Task Force
EBM: evidence-based medicine
EPC: Evidence-based Practice Center
FDA: Food and Drug Administration
GRADE: Grading of Recommendations, Assessment, Development and Evaluation
GWAS: genome-wide-association-studies
HEALM: Hierarchies of Evidence Applied to Lifestyle Medicine
HRT: hormone replacement therapy
OCEBM: Oxford Centre for Evidence-Based Medicine
PRISMA: Preferred Reporting Items for Systematic Reviews and Meta-analyses
RCT: randomized controlled trial
ROB: risk of bias
SOE: strength of evidence
THI: True Health Initiative

## References

[1] Ioannidis JP, Trepanowski JF (2018). Disclosures in nutrition research: why it is different. JAMA..

[2] Laville M, Segrestin B, Alligier M, Ruano-Rodríguez C, Serra-Majem L, Hiesmayr M (2017). Evidence-based practice within nutrition: what are the barriers for improving the evidence and how can they be dealt with?. Trials..

[3] Jørgensen T, Jacobsen RK, Toft U, Aadahl M, Glümer C, Pisinger C (2014). Effect of screening and lifestyle counselling on incidence of ischaemic heart disease in general population: Inter99 randomised trial. BMJ..

[4] Katz DLFE, Medicine FMDL (2019). Maxcy-Rosenau-last public health and preventive Medicine. 16th Ed. Production: January.

[5] Atkins D, Best D, Briss PA, Eccles M, Falck-Ytter Y, Flottorp S (2004). Grading quality of evidence and strength of recommendations. BMJ (Clinical research ed).

[6] Owens DK, Lohr KN, Atkins D, Treadwell JR, Reston JT, Bass EB (2010). AHRQ series paper 5: grading the strength of a body of evidence when comparing medical interventions—Agency for Healthcare Research and Quality and the effective health-care program. J Clin Epidemiol.

[7] Guyatt GH, Oxman AD, Schünemann HJ, Tugwell P, Knottnerus A (2011). GRADE guidelines: a new series of articles in the journal of clinical epidemiology. J Clin Epidemiol.

[8] Guyatt GH, Oxman AD, Kunz R, Vist GE, Falck-Ytter Y, Schünemann HJ (2008). Rating quality of evidence and strength of recommendations: what is “quality of evidence” and why is it important to clinicians?. BMJ: British medical journal.

[9] Guyatt GH, Oxman AD, Kunz R, Falck-Ytter Y, Vist GE, Liberati A (2008). Rating quality of evidence and strength of recommendations: going from evidence to recommendations. BMJ: British Medical Journal.

[10] Kushner RF, Sorensen KW (2013). Lifestyle medicine: the future of chronic disease management. Curr Opin Endocrinol Diabetes Obes.

[11] Ioannidis JP. We need more randomized trials in nutrition—preferably large, long-term, and with negative results. Oxford University Press. 2016. 10.3945/ajcn.116.136085 27146649

[12] Rosen L, Manor O, Engelhard D, Zucker D (2006). In defense of the randomized controlled trial for health promotion research. Am J Public Health.

[13] Willett WC (2018). Diet and health—finding a path to Veritas. Eur J Epidemiol.

[14] Barnard ND, Willett WC, Ding EL (2017). The misuse of meta-analysis in nutrition research. JAMA..

[15] Mokdad AH, Ballestros K, Echko M, Glenn S, Olsen HE, Mullany E (2018). The state of US health, 1990-2016: burden of diseases, injuries, and risk factors among US states. JAMA..

[16] Horwitz RI, Hayes-Conroy A, Caricchio R, Singer BH. From evidence based Medicine to Medicine based evidence. Am J Med. 2017.

[17] Hannan EL (2008). Randomized clinical trials and observational studies. guidelines for assessing respective strengths and limitations JACC Cardiovasc Interv.

[18] Feinstein AR, Horwitz RI (1997). Problems in the “evidence” of “evidence-based medicine”. Am J Med.

[19] Frieden TR (2017). Evidence for health decision making - beyond randomized, controlled trials. N Engl J Med.

[20] Guerin E (2012). Disentangling vitality, well-being, and quality of life: a conceptual examination emphasizing their similarities and differences with special application in the physical activity domain. J Phys Act Health.

[21] Fries J, Green L, Levine S (1989). Health promotion and the compression of morbidity. Lancet.

[22] Charlton BM, Rich-Edwards JW, Colditz GA, Missmer SA, Rosner BA, Hankinson SE (2014). Oral contraceptive use and mortality after 36 years of follow-up in the Nurses' health study: prospective cohort study. BMJ..

[23] Elliot AJ, Mooney CJ, Infurna FJ, Chapman BP (2017). Associations of lifetime trauma and chronic stress with C-reactive protein in adults ages 50 years and older: examining the moderating role of perceived control. Psychosom Med.

[24] Reinikainen J, Laatikainen T, Karvanen J, Tolonen H (2015). Lifetime cumulative risk factors predict cardiovascular disease mortality in a 50-year follow-up study in Finland. Int J Epidemiol.

[25] Ioannidis JP (2008). Some main problems eroding the credibility and relevance of randomized trials. Bull NYU Hosp Jt Dis.

[26] Seidelmann SB, Claggett B, Cheng S, Henglin M, Shah A, Steffen LM (2018). Dietary carbohydrate intake and mortality: a prospective cohort study and meta-analysis. Lancet Public Health.

[27] Crichton GE, Howe PR, Buckley JD, Coates AM, Murphy KJ, Bryan J (2012). Long-term dietary intervention trials: critical issues and challenges. Trials..

[28] The alpha-tocopherol, beta-carotene lung cancer prevention study: design, methods, participant characteristics, and compliance. The ATBC Cancer Prevention Study Group. Ann Epidemiol. 1994;4(1):1–10. 10.1016/1047-2797(94)90036-1 8205268

[29] Nakamura R (2011). Animal models and basic science—bench to bedside: session introduction. ILAR J.

[30] Sanson-Fisher RW, Bonevski B, Green LW, D'Este C (2007). Limitations of the randomized controlled trial in evaluating population-based health interventions. Am J Prev Med.

[31] Moher D, Liberati A, Tetzlaff J, Altman DG (2009). Group P. preferred reporting items for systematic reviews and meta-analyses: the PRISMA statement. Ann Intern Med.

[32] PROSPERO - International prospective register of systematic reviews [Available from: https://www.crd.york.ac.uk/prospero/].

[33] David Katz, Jonathan Fielding, Lawrence Green, Ralph Horwitz, John Ioannidis, Walter Willett, Mei Chung, Micaela Karlsen, Marissa Shams-White, Ayumi Saito, Deena Wang. Hierarchies of evidence applied to lifestyle Medicine (HEaLM): a methodological systematic review of evidence grading tools to inform development of the best method to assess strength of evidence for lifestyle medicine interventions and related clinical outcomes, including longevity and healthy aging. PROSPERO 2018 CRD42018082148 available from: https://www.crd.york.ac.uk/prospero/display_record.php?RecordID=82148.

[34] Ouzzani M, Hammady H, Fedorowicz Z, Elmagarmid A (2016). Rayyan-a web and mobile app for systematic reviews. Syst Rev.

[35] Atkins D, Best D, Briss PA, Eccles M, Falck-Ytter Y, Flottorp S (2004). Grading quality of evidence and strength of recommendations. BMJ..

[36] Food, Administration D. Guidance for industry and FDA: interim evidence-based ranking system for scientific data. July 10, 2003.

[37] Sallis JF, Prochaska JJ, Taylor WC (2000). A review of correlates of physical activity of children and adolescents. Med Sci Sports Exerc.

[38] Bully Paola, Sánchez Álvaro, Zabaleta-del-Olmo Edurne, Pombo Haizea, Grandes Gonzalo (2015). Evidence from interventions based on theoretical models for lifestyle modification (physical activity, diet, alcohol and tobacco use) in primary care settings: A systematic review. Preventive Medicine.

[39] van Poppel MN, Koes BW, Smid T, Bouter LM (1997). A systematic review of controlled clinical trials on the prevention of back pain in industry. Occup Environ Med.

[40] Slavin RE (1995). Best evidence synthesis: an intelligent alternative to meta-analysis. J Clin Epidemiol.

[41] Mansi S, Milosavljevic S, Baxter GD, Tumilty S, Hendrick P (2014). A systematic review of studies using pedometers as an intervention for musculoskeletal diseases. BMC Musculoskelet Disord.

[42] Geraedts H, Zijlstra A, Bulstra SK, Stevens M, Zijlstra W (2013). Effects of remote feedback in home-based physical activity interventions for older adults: a systematic review. Patient Educ Couns.

[43] Singh A, Uijtdewilligen L, Twisk JW, Van Mechelen W, Chinapaw MJ (2012). Physical activity and performance at school: a systematic review of the literature including a methodological quality assessment. Arch Pediatr Adolesc Med.

[44] Peurala SH, Karttunen AH, Sjögren T, Paltamaa J, Heinonen A (2014). Evidence for the effectiveness of walking training on walking and self-care after stroke: a systematic review and meta-analysis of randomized controlled trials. J Rehabil Med.

[45] Stuck AE, Walthert JM, Nikolaus T, Bula CJ, Hohmann C, Beck JC (1999). Risk factors for functional status decline in community-living elderly people: a systematic literature review. Soc Sci Med.

[46] van der Vorst A, Zijlstra GR, De Witte N, Duppen D, Stuck AE, Kempen GI (2016). Limitations in activities of daily living in community-dwelling people aged 75 and over: a systematic literature review of risk and protective factors. PLoS One.

[47] Briss PA, Zaza S, Pappaioanou M, Fielding J, Wright-De Aguero L, Truman BI (2000). Developing an evidence-based guide to community preventive services--methods. The task force on community preventive services. Am J Prev Med.

[48] Moyer V, Bibbins-Domingo K (2015). The US preventive services task force: what is it and what does it do?. N C Med J.

[49] Scientific Report of the 2015 Dietary Guidelines Advisory Committee. 2015.

[50] Handu D, Moloney L, Wolfram T, Ziegler P, Acosta A, Steiber A (2016). Academy of nutrition and dietetics methodology for conducting systematic reviews for the evidence analysis library. J Acad Nutr Diet.

[51] Berkman ND, Lohr KN, Ansari MT, Balk EM, Kane R, McDonagh M (2015). Grading the strength of a body of evidence when assessing health care interventions: an EPC update. J Clin Epidemiol.

[52] Institute JB. New JBI levels of Evidence 2013. Available from: https://www.google.com/url?client=internal-uds-cse&cx=007368958558683417275:2imkdaua2-c&q=https://joannabriggs.org/sites/default/files/2019-05/JBI%2520Levels%2520of%2520Evidence%2520Supporting%2520Documents-v2.pdf&sa=U&ved=2ahUKEwiWppTP2P3jAhVSHqwKHdlRDRgQFjACegQIDxAB&usg=AOvVaw0bFx9zTocvgNC_em53nIBf.

[53] Oxford Center for Evidence-Based Medicine Levels of Evidence. Accessed online May Afhwcno-l-o-e.

[54] Guidelines ACoCAHATFoP. Methodology Manual and Policies From the ACCF/AHA Task Force on Practice Guidelines2010 May 2018. Available from: http://professional.heart.org/professional/GuidelinesStatements/PublicationDevelopment/UCM_320470_Methodologies-and-Policies-from-the-ACCAHA-Task-Force-on-Practice-Guidelines.jsp.

[55] Katz DL, Meller S (2014). Can we say what diet is best for health?. Annu Rev Public Health.

[56] Blake P, Durao S, Naude CE, Bero L (2018). An analysis of methods used to synthesize evidence and grade recommendations in food-based dietary guidelines. Nutr Rev.

[57] National Academies of Sciences E, Medicine. Guiding principles for developing dietary reference intakes based on chronic disease: National Academies Press; 2017. 29200241

[58] Global, regional, and national age-sex-specific mortality for 282 causes of death in 195 countries and territories, 1980–2017: a systematic analysis for the Global Burden of Disease Study 2017. Lancet. 2018;392(10159):1736–1788. 10.1016/S0140-6736(18)32203-7 PMC6227606 30496103

[59] Belbasis L, Köhler C, Stefanis N, Stubbs B, van Os J, Vieta E (2018). Risk factors and peripheral biomarkers for schizophrenia spectrum disorders: an umbrella review of meta-analyses. Acta Psychiatr Scand.

[60] Manson JE, Chlebowski RT, Stefanick ML, Aragaki AK, Rossouw JE, Prentice RL (2013). Menopausal hormone therapy and health outcomes during the intervention and extended poststopping phases of the Women’s Health Initiative randomized trials. JAMA..

[61] Anglemyer A, Horvath HT, Bero L. Healthcare outcomes assessed with observational study designs compared with those assessed in randomized trials. Cochrane Database Syst Rev. (2014, 4):MR000034. 10.1002/14651858.MR000034.pub2 PMC8191367 24782322

[62] Estruch R, Ros E, Salas-Salvado J, Covas MI, Corella D, Aros F (2018). Primary prevention of cardiovascular disease with a Mediterranean diet supplemented with extra-virgin olive oil or nuts. N Engl J Med.

[63] Goldberg RB, Bray GA, Marcovina SM, Mather KJ, Orchard TJ, Perreault L, et al. Non-traditional biomarkers and incident diabetes in the diabetes prevention program: comparative effects of lifestyle and metformin interventions. Diabetologia. 2018. 10.1007/s00125-018-4748-2 PMC6456055 30334082

[64] Sarrel PM, Njike VY, Vinante V, Katz DL (2013). The mortality toll of estrogen avoidance: an analysis of excess deaths among hysterectomized women aged 50 to 59 years. Am J Public Health.

[65] Prentice RL, Langer R, Stefanick ML, Howard BV, Pettinger M, Anderson G (2005). Combined postmenopausal hormone therapy and cardiovascular disease: toward resolving the discrepancy between observational studies and the Women's Health Initiative clinical trial. Am J Epidemiol.

[66] Hodis HN, Sarrel PM (2018). Menopausal hormone therapy and breast cancer: what is the evidence from randomized trials?. Climacteric : the journal of the International Menopause Society.

[67] Silverman SL (2009). From randomized controlled trials to observational studies. Am J Med.

