# Supplementary material for: Hierarchies of evidence applied to lifestyle Medicine (HEALM): introduction of a strength-of-evidence approach based on a methodological systematic review
Source: BMC Med Res Methodol. 2019 Aug 20;19:178. doi: 10.1186/s12874-019-0811-z (PMC6701153; doi:10.1186/s12874-019-0811-z)
Supplement: Supplementary file 1 — Expert panel (DOCX 13 kb) [file 12874_2019_811_MOESM1_ESM.docx]

Additional file 1: Expert Panel

**Name:** Mei Chung
**Affiliation:** Department of Public Health and Community Medicine, School of Medicine, Tufts University
**Expertise:** Nutrition and health policy, clinical practice guidelines, systematic reviews, meta-analysis, nutritional epidemiology


**Name:** Lawrence Green
**Affiliation:** Department of Epidemiology and Biostatistics, University of California at San Francisco School of Medicine
**Expertise:** Epidemiology and biostatistics

**Name:** Jonathan Fielding
**Affiliation:** UCLA Fielding School of Public Health
**Expertise:** Health policy and management, preventative medicine, public health

**Name:** Walter Willett
**Affiliation:** Harvard University T.H. Chan School of Public Health
**Expertise:** Nutritional epidemiology and methodology
