# Supplementary material for: Hierarchies of evidence applied to lifestyle Medicine (HEALM): introduction of a strength-of-evidence approach based on a methodological systematic review
Source: BMC Med Res Methodol. 2019 Aug 20;19:178. doi: 10.1186/s12874-019-0811-z (PMC6701153; doi:10.1186/s12874-019-0811-z)
Supplement: Supplementary file 2 — Search strategy (DOCX 17 kb) [file 12874_2019_811_MOESM2_ESM.docx]

Additional file 2: Search Strategy

**PubMed Longevity**

“aging well” [text word] OR longevity [MeSH] OR “healthy aging” [text word] OR “successful aging’ [text word]

AND

Filters: Systematic Reviews OR Meta-Analysis

AND

Limits: last 5 years, Humans

**PubMed Lifestyle**

diet [MeSH] or nutritional status [MeSH] or stress, psychological [MeSH] or exercise [MeSH] or social support [MeSH] or family relations [MeSH] or social isolation [MeSH] or substance related disorders [MeSH] or sleep [MeSH]

NOT

Epidemiologic studies [MeSH] or “in vitro” [text word] or stem cell [text word] or gwas [text word] or genetic [text word]

NOT

Mouse [text word] or murine [text word] or rat [text word] or pig [text word] or porcine [text word] or horse [text word] or equine [text word] or cow [text word] or bovine [text word] or cat [text word] or feline [text word] or dog [text word] or canine [test word] or amphibian [text word] or guinea pig [text word] or reptile [text word]

Meta analysis or systematic review

5 years

Aged 65 +

**Embase Longevity**

#6 ('longevity'/exp OR 'healthy aging'/exp OR 'successful aging'/exp OR 'healthy aging' OR 'successful aging') AND ([cochrane review]/lim OR [systematic review]/lim OR [meta analysis]/lim) AND [embase]/lim AND ([article]/lim OR [article in press]/lim) AND [humans]/lim AND [2013-2017]/py **86**

#5 ('longevity'/exp OR 'healthy aging'/exp OR 'successful aging'/exp OR 'healthy aging' OR 'successful aging') AND ([cochrane review]/lim OR [systematic review]/lim OR [meta analysis]/lim) AND [embase]/lim AND ([article]/lim OR [article in press]/lim) AND [humans]/lim **120**

#4 ('longevity'/exp OR 'healthy aging'/exp OR 'successful aging'/exp OR 'healthy aging' OR 'successful aging') AND ([cochrane review]/lim OR [systematic review]/lim OR [meta analysis]/lim) AND [embase]/lim AND ([article]/lim OR [article in press]/lim) **124**

#3 ('longevity'/exp OR 'healthy aging'/exp OR 'successful aging'/exp OR 'healthy aging' OR 'successful aging') AND ([cochrane review]/lim OR [systematic review]/lim OR [meta analysis]/lim) AND [embase]/lim **319**

#2 ('longevity'/exp OR 'healthy aging'/exp OR 'successful aging'/exp OR 'healthy aging' OR 'successful aging') AND ([cochrane review]/lim OR [systematic review]/lim OR [meta analysis]/lim) **359**

#1 'longevity'/exp OR 'healthy aging'/exp OR 'successful aging'/exp OR 'healthy aging' OR 'successful aging' 32,493

**Embase Lifestyle**

#13

('diet'/exp OR 'nutritional status'/exp OR 'mental stress'/exp OR 'sleep quality'/exp OR 'physical activity, capacity and performance'/exp OR 'social support'/exp OR 'social network'/exp OR 'psychological well-being'/exp OR 'family relationships'/exp OR 'family relation'/exp OR 'addiction'/exp) AND [2013-2017]/py AND ([cochrane review]/lim OR [systematic review]/lim OR [meta analysis]/lim) AND ([article]/lim OR [article in press]/lim) AND [embase]/lim AND [humans]/lim AND [aged]/lim **123**

#12

('diet'/exp OR 'nutritional status'/exp OR 'mental stress'/exp OR 'sleep quality'/exp OR 'physical activity, capacity and performance'/exp OR 'social support'/exp OR 'social network'/exp OR 'psychological well-being'/exp OR 'family relationships'/exp OR 'family relation'/exp OR 'addiction'/exp) AND [2013-2017]/py AND ([cochrane review]/lim OR [systematic review]/lim OR [meta analysis]/lim) AND ([article]/lim OR [article in press]/lim) AND [embase]/lim AND [humans]/lim **3,269**

#11

('diet'/exp OR 'nutritional status'/exp OR 'mental stress'/exp OR 'sleep quality'/exp OR 'physical activity, capacity and performance'/exp OR 'social support'/exp OR 'social network'/exp OR 'psychological well-being'/exp OR 'family relationships'/exp OR 'family relation'/exp OR 'addiction'/exp) AND [2013-2017]/py AND ([cochrane review]/lim OR [systematic review]/lim OR [meta analysis]/lim) AND ([article]/lim OR [article in press]/lim) AND [embase]/lim **3,310**

#10

('diet'/exp OR 'nutritional status'/exp OR 'mental stress'/exp OR 'sleep quality'/exp OR 'physical activity, capacity and performance'/exp OR 'social support'/exp OR 'social network'/exp OR 'psychological well-being'/exp OR 'family relationships'/exp OR 'family relation'/exp OR 'addiction'/exp) AND [2013-2017]/py AND ([cochrane review]/lim OR [systematic review]/lim OR [meta analysis]/lim) AND ([article]/lim OR [article in press]/lim) **4,142**

#9

('diet'/exp OR 'nutritional status'/exp OR 'mental stress'/exp OR 'sleep quality'/exp OR 'physical activity, capacity and performance'/exp OR 'social support'/exp OR 'social network'/exp OR 'psychological well-being'/exp OR 'family relationships'/exp OR 'family relation'/exp OR 'addiction'/exp) AND [2013-2017]/py AND ([cochrane review]/lim OR [systematic review]/lim OR [meta analysis]/lim) **12,015**

#8

('diet'/exp OR 'nutritional status'/exp OR 'mental stress'/exp OR 'sleep quality'/exp OR 'physical activity, capacity and performance'/exp OR 'social support'/exp OR 'social network'/exp OR 'psychological well-being'/exp OR 'family relationships'/exp OR 'family relation'/exp OR 'addiction'/exp) AND [2013-2017]/py **462,481**

#7

'diet'/exp OR 'nutritional status'/exp OR 'mental stress'/exp OR 'sleep quality'/exp OR 'physical activity, capacity and performance'/exp OR 'social support'/exp OR 'social network'/exp OR 'psychological well-being'/exp OR 'family relationships'/exp OR 'family relation'/exp OR 'addiction'/exp **1,532,652**

**AnthropologyPlus**

Metaanalysis OR meta analysis OR metasynthesis or Meta synthesis or systematic review

2013 - 2017

**PsychInfo Longevity**

AB, TI (ag?ing well) or AB, TI (healthy ag?ing) or AB, TI (successful ag?ing) OR SU (life expectancy) or AB, TI (longevity)

Systematic review or meta analysis or metasynthesis

Journal or peer reviewed journal or journal article or scholarly journals

2013 🡪

**PsychInfo Lifestyle**

SU (exp diets) or SU (exp psychological stress) or SU (exp social support) or SU (exp exercise) or SU (exp social isolation) or SU (exp drug abuse) or SU (sleep) or AB, TI (nutritional status)

Systematic review or meta analysis or metasynthesis

Journal or peer reviewed journal or journal article

Aged 65 + or very old 85+

2013 ->

**Ageline Longevity**

Healthy aging or successful aging or longevity or aging well AND

Meta-analysis or metaanalysis or meta-synthesis or metasynthesis or systematic review

2012 -2017

Journal article

**Ageline Lifestyle**

Diet or nutritional status or stress or exercise or social support or family relation* or isolation or substance abuse or sleep

AND

Meta-synthesis or metasynthesis or meta-analysis or metaanalysis or systematic review or systematic literature review

2013 - 2017

Academic journals
