# Supplementary material for: Hierarchies of evidence applied to lifestyle Medicine (HEALM): introduction of a strength-of-evidence approach based on a methodological systematic review
Source: BMC Med Res Methodol. 2019 Aug 20;19:178. doi: 10.1186/s12874-019-0811-z (PMC6701153; doi:10.1186/s12874-019-0811-z)
Supplement: Supplementary file 3 — Keywords used in text mining (DOCX 13 kb) [file 12874_2019_811_MOESM3_ESM.docx]

Additional file 3: Keywords Used In Text Mining

1. Grading of Recommendations, Assessment, Development and Evaluation

2. GRADE approach

3. grade

4. graded

5. grading

6. soe

7. quality of evidence/quality of the evidence

8. levels of evidence

9. rated

10. inadequate evidence

11. strong evidence

12. moderate evidence

13. low evidence/limited evidence

14. insufficient evidence

15. rating

16. methodological quality

17. strength of evidence
